# *O*-Phenyl (*tert*-butyl­amido)(*p*-tolyl­amido)­phosphinate

**DOI:** 10.1107/S1600536811048537

**Published:** 2011-11-23

**Authors:** Mehrdad Pourayoubi, Arnold L. Rheingold, Chao Chen, Fatemeh Karimi Ahmadabad, Atekeh Tarahhomi

**Affiliations:** a Department of Chemistry, Ferdowsi University of Mashhad, Mashhad, Iran; b Department of Chemistry, University of California, San Diego, 9500 Gilman Drive, La Jolla, CA 92093, USA

## Abstract

In the title mol­ecule, C_17_H_23_N_2_O_2_P, the P atom has a distorted tetra­hedral environment. The P—N bond to the tolyl­amido fragment is 1.642 (4) Å while that to the butyl­amido fragment is 1.629 (3) Å. The dihedral angle between the two benzene rings is 82.3 (2)°. In the crystal, adjacent mol­ecules are linked *via* weak N—H⋯(O)P and N—H⋯N hydrogen-bonding inter­actions into an extended chain parallel to the *b* axis. The three methyl groups of the *tert*-butyl­amido substituent are disordered over two sets of sites with equal occupancies. The crystal studied was found to be a non-merohedral twin with the minor twin component = 23.1 (1)%.

## Related literature

For background to mixed-amido phosphinates, see: Pourayoubi *et al.* (2011*a* ▶); Sabbaghi *et al.* (2011 ▶). For the *sp* ^2^ character of the nitro­gen atom of the P(=O)N unit and also for its low Lewis-base character in acting as a hydrogen-bond acceptor, see: Toghraee *et al.* (2011 ▶); Pourayoubi *et al.* (2011*b* ▶,*c* ▶). For a description of the Cambridge Structure Database, see: Allen (2002 ▶).
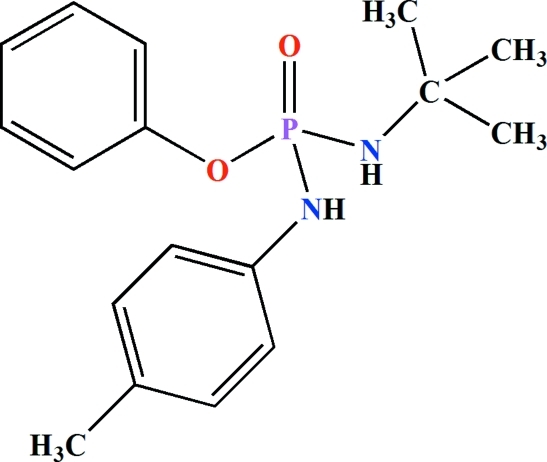


## Experimental

### 

#### Crystal data

C_17_H_23_N_2_O_2_P
*M* *_r_* = 318.34
Monoclinic, 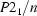

*a* = 11.412 (5) Å
*b* = 9.519 (4) Å
*c* = 15.768 (6) Å
β = 104.332 (5)°
*V* = 1659.5 (12) Å^3^
*Z* = 4
Mo *K*α radiation
μ = 0.18 mm^−1^
*T* = 100 K
0.20 × 0.18 × 0.15 mm

#### Data collection

Bruker APEX CCD diffractometer
Absorption correction: multi-scan (*TWINABS*; Sheldrick, 2008*a* ▶) *T*_min_ = 0.966, *T*_max_ = 0.974
18075 measured reflections
3860 independent reflections
2497 reflections with *I* > 2σ(*I*)
*R*_int_ = 0.087

#### Refinement

*R*[*F* ^2^ > 2σ(*F* ^2^)] = 0.089
*wR*(*F* ^2^) = 0.203
*S* = 1.08
3860 reflections
235 parameters
H-atom parameters constrained
Δρ_max_ = 0.36 e Å^−3^
Δρ_min_ = −0.42 e Å^−3^

### 

Data collection: *APEX2* (Bruker, 2005 ▶); cell refinement: *SAINT* (Bruker, 2005 ▶); data reduction: *CELL_NOW* (Sheldrick, 2008*a* ▶) and *SAINT*; program(s) used to solve structure: *SHELXS97* (Sheldrick, 2008*b* ▶); program(s) used to refine structure: *SHELXL97* (Sheldrick, 2008*b* ▶); molecular graphics: *Mercury* (Macrae *et al.*, 2008 ▶); software used to prepare material for publication: *SHELXTL* (Sheldrick, 2008*b* ▶) and *enCIFer* (Allen *et al.*, 2004 ▶).

## Supplementary Material

Crystal structure: contains datablock(s) global, I. DOI: 10.1107/S1600536811048537/wm2550sup1.cif

Structure factors: contains datablock(s) I. DOI: 10.1107/S1600536811048537/wm2550Isup2.hkl

Additional supplementary materials:  crystallographic information; 3D view; checkCIF report

## Figures and Tables

**Table 1:** Hydrogen-bond geometry (Å, °)

*D*—H⋯*A*	*D*—H	H⋯*A*	*D*⋯*A*	*D*—H⋯*A*
N1—H1⋯N2^i^	0.88	2.32	3.175 (5)	163
N2—H2⋯O1^ii^	0.88	2.40	3.275 (5)	170

Symmetry codes: (i) 
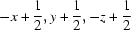
; (ii) 
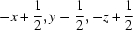
.

**Fig. 1.**
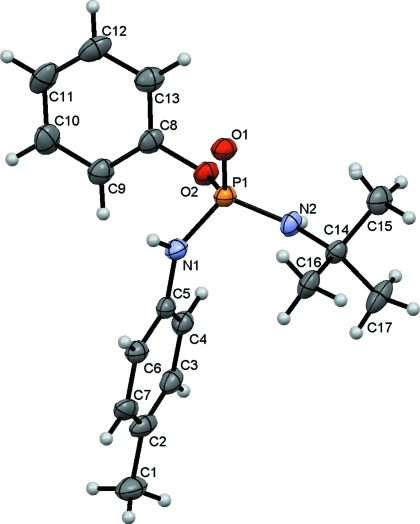
An ORTEP-style plot and atom labeling scheme for the title compound. Displacement ellipsoids are given at 50% probability level and H atoms are drawn as small spheres of arbitrary radius. The disorder of the methyl groups is not shown.

**Fig. 2.**
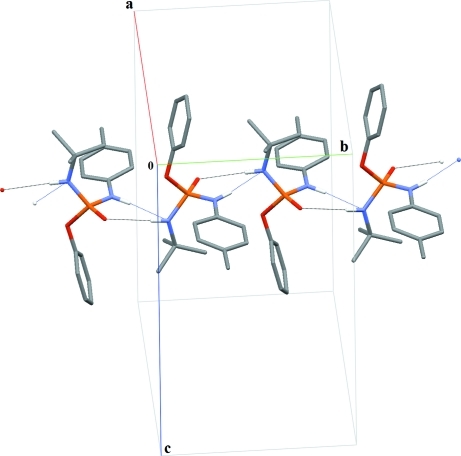
Partial packing view showing the formation of the chain through the N—H···(O)P and N—H···N hydrogen bonds which are shown as dashed lines. The H atoms not involved in hydrogen bonding have been omitted for the sake of clarity.

## supplementary crystallographic information

### Comment

Following our previous work on the synthesis of mixed-amido phosphinates containing a P(O)(O)(NH)(NH) skeleton (Pourayoubi *et al.*, 2011*a*), we report here on the synthesis and crystal structure of the title compound, P(O)[OC_6_H_5_][NHC_6_H_4_(4-CH_3_)][NHC(CH_3_)_3_] (Fig. 1).

The P═O, P—O and P—N bond lengths and P—O—C and P—N—C bond angles are within the expected values (Sabbaghi *et al.*, 2011). The P atom has a distorted tetrahedral conformation with the bond angles in the range of 96.76 (17)° [O2–P1–N2] to 117.03 (17)° [O1–P1–N2]. The P1—N1 bond (with length of 1.642 (4) Å) is slightly longer than the P1—N2 bond (1.629 (3) Å). The dihedral angle between the phenyl rings of the OC_6_H_5_ and NHC_6_H_4_(4-CH_3_) moieties is 82.3 (2)°.

In the crystal structure, the molecules are linked by weak N—H···(O)P and N—H···N hydrogen bonding interactions (Table 1) into an extended chain along [010] (Fig. 2). As illustrated for phosphoramidates by Toghraee *et al.* (2011) and Pourayoubi *et al.* (2011*b*,*c*) by examining all deposited phosphoramidates in the Cambridge Structural Database (CSD, Version 5.32, May 2011 update; Allen, 2002), the N atom of the P(═O)N unit usually adopts an *sp*^2^ character (which is reflected in the bond angles at the N atom) and usually does not act as an acceptor in hydrogen bonding interactions. Therefore, the N—H···N—P contact in the crystal packing may rather be attributed to the assembly of the molecules with respect to one another.

### Experimental

To a solution of (C_6_H_5_O)(4-CH_3_C_6_H_4_NH)P(O)Cl (1.714 mmol) in chloroform, a solution of *tert*-butylamine (3.428 mmol) in chloroform was added at 273 K. After 5 h stirring, the solvent was removed in vacuum and the solid product was washed with distilled water. Single crystals were obtained from a mixture of CH_3_CN/CHCl_3_ at room temperature.

### Refinement

The investigated crystal was found to be a two-component rotational twin. The data for both components were integrated using *SAINT* and scaled with *TWINABS*. Final refinement was done using a *HKLF5* file generated by *TWINABS* with an appropriate *BASF* parameter (0.23089 (10)). The three methyl groups of the *tert*-butyl moiety were refined as being disordered in a 0.5:0.5 ratio. All H atoms were placed geometrically using a riding model. Their positions were constrained relative to their parent atom using the appropriate HFIX command in *SHELXL97* (d(C—H) = 0.98 Å for methyl H atoms, d(C—H) = 0.95 Å for aromatic H atoms and d(N—H) = 0.88 Å for amide H atoms, with U_iso_(H) = 1.2U_eq_(C,N) for aromatic and amide H atoms and U_iso_(H) = 1.5U_eq_(C) for methyl H atoms).

### Crystal data

**Table:**

C_17_H_23_N_2_O_2_P	*F*(000) = 680
*M**_r_* = 318.34	*D*_x_ = 1.274 Mg m^−^^3^
Monoclinic, *P*2_1_/*n*	Mo *K*α radiation, λ = 0.71073 Å
Hall symbol: -P 2yn	Cell parameters from 2700 reflections
*a* = 11.412 (5) Å	θ = 2.5–27.3°
*b* = 9.519 (4) Å	µ = 0.18 mm^−^^1^
*c* = 15.768 (6) Å	*T* = 100 K
β = 104.332 (5)°	Block, colourless
*V* = 1659.5 (12) Å^3^	0.20 × 0.18 × 0.15 mm
*Z* = 4	

### Data collection

**Table:**

Bruker APEX CCD diffractometer	18075 independent reflections
Radiation source: fine-focus sealed tube	2497 reflections with *I* > 2σ(*I*)
graphite	*R*_int_ = 0.087
φ and ω scans	θ_max_ = 28.3°, θ_min_ = 2.0°
Absorption correction: multi-scan (*TWINABS*; Sheldrick, 2008*a*)	*h* = −15→14
*T*_min_ = 0.966, *T*_max_ = 0.974	*k* = 0→12
3860 measured reflections	*l* = 0→20

### Refinement

**Table:**

Refinement on *F*^2^	Secondary atom site location: difference Fourier map
Least-squares matrix: full	Hydrogen site location: inferred from neighbouring sites
*R*[*F*^2^ > 2σ(*F*^2^)] = 0.089	H-atom parameters constrained
*wR*(*F*^2^) = 0.203	*w* = 1/[σ^2^(*F*_o_^2^) + (0.032*P*)^2^ + 5.6384*P*] where *P* = (*F*_o_^2^ + 2*F*_c_^2^)/3
*S* = 1.08	(Δ/σ)_max_ < 0.001
3860 reflections	Δρ_max_ = 0.36 e Å^−^^3^
235 parameters	Δρ_min_ = −0.42 e Å^−^^3^
0 restraints	Extinction correction: *SHELXL97* (Sheldrick, 2008*b*), Fc^*^=kFc[1+0.001xFc^2^λ^3^/sin(2θ)]^-1/4^
Primary atom site location: structure-invariant direct methods	Extinction coefficient: 0.0051 (8)

### Special details

**Table:**

Geometry. All e.s.d.'s (except the e.s.d. in the dihedral angle between two l.s. planes) are estimated using the full covariance matrix. The cell e.s.d.'s are taken into account individually in the estimation of e.s.d.'s in distances, angles and torsion angles; correlations between e.s.d.'s in cell parameters are only used when they are defined by crystal symmetry. An approximate (isotropic) treatment of cell e.s.d.'s is used for estimating e.s.d.'s involving l.s. planes.
Refinement. Refinement of *F*^2^ against ALL reflections. The weighted *R*-factor *wR* and goodness of fit *S* are based on *F*^2^, conventional *R*-factors *R* are based on *F*, with *F* set to zero for negative *F*^2^. The threshold expression of *F*^2^ > σ(*F*^2^) is used only for calculating *R*-factors(gt) *etc*. and is not relevant to the choice of reflections for refinement. *R*-factors based on *F*^2^ are statistically about twice as large as those based on *F*, and *R*- factors based on ALL data will be even larger.

### Fractional atomic coordinates and isotropic or equivalent isotropic displacement parameters (Å^2^)

**Table:**

	*x*	*y*	*z*	*U*_iso_*/*U*_eq_	Occ. (<1)
C1	−0.3828 (4)	0.3138 (7)	0.1636 (3)	0.0484 (14)	
H1A	−0.4241	0.3213	0.1014	0.073*	
H1B	−0.4038	0.3950	0.1951	0.073*	
H1C	−0.4083	0.2273	0.1876	0.073*	
C2	−0.2482 (4)	0.3104 (6)	0.1738 (3)	0.0375 (11)	
C3	−0.1876 (4)	0.1854 (5)	0.1663 (2)	0.0330 (10)	
H3	−0.2329	0.1008	0.1548	0.040*	
C4	−0.0631 (4)	0.1802 (5)	0.1750 (2)	0.0293 (9)	
H4	−0.0249	0.0933	0.1688	0.035*	
C5	0.0049 (3)	0.3026 (5)	0.1927 (2)	0.0264 (9)	
C6	−0.0545 (3)	0.4307 (5)	0.1998 (2)	0.0288 (9)	
H6	−0.0095	0.5155	0.2113	0.035*	
C7	−0.1785 (4)	0.4320 (5)	0.1901 (2)	0.0338 (10)	
H7	−0.2174	0.5189	0.1948	0.041*	
C8	0.2197 (4)	0.1078 (5)	0.0673 (3)	0.0323 (10)	
C9	0.1197 (4)	0.1458 (5)	0.0015 (3)	0.0362 (11)	
H9	0.0411	0.1470	0.0118	0.043*	
C10	0.1373 (5)	0.1821 (6)	−0.0801 (3)	0.0449 (12)	
H10	0.0700	0.2084	−0.1261	0.054*	
C11	0.2522 (5)	0.1802 (6)	−0.0946 (3)	0.0450 (12)	
H11	0.2638	0.2068	−0.1500	0.054*	
C12	0.3495 (5)	0.1395 (6)	−0.0283 (3)	0.0439 (12)	
H12	0.4277	0.1364	−0.0391	0.053*	
C13	0.3357 (4)	0.1030 (5)	0.0541 (3)	0.0369 (11)	
H13	0.4031	0.0757	0.0998	0.044*	
C14	0.2211 (4)	0.1101 (5)	0.3984 (2)	0.0286 (9)	
C15	0.3531 (8)	0.0707 (13)	0.4489 (6)	0.041 (2)	0.50
H15A	0.3657	−0.0303	0.4432	0.061*	0.50
H15B	0.3653	0.0948	0.5109	0.061*	0.50
H15C	0.4108	0.1231	0.4242	0.061*	0.50
C16	0.1961 (10)	0.2594 (11)	0.4144 (6)	0.038 (2)	0.50
H16A	0.2495	0.3199	0.3905	0.058*	0.50
H16B	0.2105	0.2757	0.4775	0.058*	0.50
H16C	0.1116	0.2811	0.3858	0.058*	0.50
C17	0.1378 (10)	0.0128 (12)	0.4394 (6)	0.043 (2)	0.50
H17A	0.0529	0.0386	0.4154	0.065*	0.50
H17B	0.1588	0.0247	0.5031	0.065*	0.50
H17C	0.1500	−0.0855	0.4251	0.065*	0.50
C15'	0.3377 (8)	0.1837 (13)	0.4350 (5)	0.041 (2)	0.50
H15D	0.4046	0.1256	0.4264	0.062*	0.50
H15E	0.3473	0.2002	0.4977	0.062*	0.50
H15F	0.3378	0.2739	0.4050	0.062*	0.50
C16'	0.1147 (9)	0.2038 (12)	0.4044 (6)	0.042 (2)	0.50
H16D	0.1245	0.2966	0.3802	0.063*	0.50
H16E	0.1123	0.2135	0.4658	0.063*	0.50
H16F	0.0391	0.1613	0.3710	0.063*	0.50
C17'	0.2138 (9)	−0.0251 (11)	0.4441 (5)	0.034 (2)	0.50
H17D	0.1421	−0.0773	0.4126	0.051*	0.50
H17E	0.2080	−0.0063	0.5040	0.051*	0.50
H17F	0.2865	−0.0810	0.4457	0.051*	0.50
N1	0.1321 (3)	0.3055 (4)	0.2034 (2)	0.0272 (8)	
H1	0.1619	0.3882	0.1951	0.033*	
N2	0.2060 (3)	0.0744 (4)	0.3037 (2)	0.0281 (8)	
H2	0.1806	−0.0109	0.2871	0.034*	
O1	0.3536 (2)	0.2436 (3)	0.24577 (18)	0.0325 (7)	
O2	0.2020 (2)	0.0677 (3)	0.14980 (17)	0.0304 (7)	
P1	0.23229 (9)	0.17878 (13)	0.22865 (6)	0.0262 (3)	

### Atomic displacement parameters (Å^2^)

**Table:**

	*U*^11^	*U*^22^	*U*^33^	*U*^12^	*U*^13^	*U*^23^
C1	0.028 (2)	0.083 (4)	0.036 (2)	−0.002 (3)	0.0118 (19)	−0.001 (3)
C2	0.024 (2)	0.070 (4)	0.0189 (19)	−0.006 (2)	0.0061 (15)	−0.004 (2)
C3	0.029 (2)	0.048 (3)	0.0203 (19)	−0.008 (2)	0.0044 (16)	0.003 (2)
C4	0.028 (2)	0.037 (2)	0.0229 (19)	−0.0024 (19)	0.0063 (15)	0.0036 (19)
C5	0.0230 (19)	0.040 (2)	0.0163 (17)	0.0016 (18)	0.0043 (14)	0.0045 (17)
C6	0.024 (2)	0.041 (3)	0.0214 (19)	−0.0008 (18)	0.0050 (15)	−0.0012 (18)
C7	0.029 (2)	0.054 (3)	0.0192 (19)	0.008 (2)	0.0083 (16)	−0.003 (2)
C8	0.038 (2)	0.038 (3)	0.022 (2)	−0.001 (2)	0.0106 (17)	−0.0050 (19)
C9	0.036 (2)	0.048 (3)	0.025 (2)	0.000 (2)	0.0081 (17)	−0.004 (2)
C10	0.057 (3)	0.053 (3)	0.024 (2)	0.010 (3)	0.009 (2)	−0.003 (2)
C11	0.063 (3)	0.051 (3)	0.027 (2)	−0.002 (3)	0.022 (2)	−0.008 (2)
C12	0.048 (3)	0.055 (3)	0.035 (2)	−0.007 (2)	0.023 (2)	−0.011 (2)
C13	0.034 (2)	0.045 (3)	0.033 (2)	−0.004 (2)	0.0097 (19)	−0.008 (2)
C14	0.031 (2)	0.037 (3)	0.0199 (19)	0.0019 (19)	0.0097 (16)	−0.0009 (18)
C15	0.038 (5)	0.063 (7)	0.019 (4)	0.001 (5)	0.002 (4)	−0.002 (5)
C16	0.047 (6)	0.049 (6)	0.023 (4)	0.000 (5)	0.015 (4)	0.006 (4)
C17	0.057 (7)	0.052 (7)	0.023 (5)	−0.018 (6)	0.016 (5)	0.001 (4)
C15'	0.040 (5)	0.066 (7)	0.019 (4)	−0.019 (5)	0.009 (4)	−0.009 (5)
C16'	0.041 (5)	0.063 (7)	0.024 (4)	0.021 (5)	0.012 (4)	−0.004 (4)
C17'	0.040 (5)	0.046 (6)	0.019 (4)	0.002 (5)	0.011 (4)	0.004 (4)
N1	0.0212 (16)	0.038 (2)	0.0229 (16)	−0.0022 (15)	0.0067 (13)	0.0015 (15)
N2	0.0318 (18)	0.035 (2)	0.0169 (16)	−0.0020 (16)	0.0045 (13)	−0.0002 (14)
O1	0.0238 (14)	0.0463 (19)	0.0275 (15)	−0.0002 (13)	0.0067 (11)	−0.0013 (14)
O2	0.0316 (15)	0.0385 (18)	0.0216 (14)	−0.0021 (13)	0.0079 (11)	−0.0046 (13)
P1	0.0215 (5)	0.0383 (6)	0.0184 (5)	0.0005 (5)	0.0045 (4)	−0.0014 (5)

### Geometric parameters (Å, °)

**Table:**

C1—C2	1.506 (6)	C14—C17'	1.488 (10)
C1—H1A	0.9800	C14—N2	1.500 (5)
C1—H1B	0.9800	C14—C16'	1.528 (10)
C1—H1C	0.9800	C14—C15	1.565 (10)
C2—C7	1.391 (7)	C14—C17	1.576 (10)
C2—C3	1.396 (7)	C15—H15A	0.9800
C3—C4	1.394 (5)	C15—H15B	0.9800
C3—H3	0.9500	C15—H15C	0.9800
C4—C5	1.390 (6)	C16—H16A	0.9800
C4—H4	0.9500	C16—H16B	0.9800
C5—C6	1.413 (6)	C16—H16C	0.9800
C5—N1	1.419 (5)	C17—H17A	0.9800
C6—C7	1.386 (5)	C17—H17B	0.9800
C6—H6	0.9500	C17—H17C	0.9800
C7—H7	0.9500	C15'—H15D	0.9800
C8—C9	1.386 (6)	C15'—H15E	0.9800
C8—C13	1.391 (6)	C15'—H15F	0.9800
C8—O2	1.417 (5)	C16'—H16D	0.9800
C9—C10	1.395 (6)	C16'—H16E	0.9800
C9—H9	0.9500	C16'—H16F	0.9800
C10—C11	1.385 (7)	C17'—H17D	0.9800
C10—H10	0.9500	C17'—H17E	0.9800
C11—C12	1.379 (7)	C17'—H17F	0.9800
C11—H11	0.9500	N1—P1	1.642 (4)
C12—C13	1.392 (6)	N1—H1	0.8800
C12—H12	0.9500	N2—P1	1.629 (3)
C13—H13	0.9500	N2—H2	0.8800
C14—C16	1.483 (11)	O1—P1	1.478 (3)
C14—C15'	1.487 (10)	O2—P1	1.603 (3)
			
C2—C1—H1A	109.5	N2—C14—C16'	107.3 (4)
C2—C1—H1B	109.5	C16—C14—C15	110.2 (7)
H1A—C1—H1B	109.5	C17'—C14—C15	73.1 (6)
C2—C1—H1C	109.5	N2—C14—C15	108.1 (4)
H1A—C1—H1C	109.5	C16'—C14—C15	142.0 (6)
H1B—C1—H1C	109.5	C16—C14—C17	109.4 (6)
C7—C2—C3	116.9 (4)	C15'—C14—C17	133.6 (6)
C7—C2—C1	121.5 (5)	N2—C14—C17	110.0 (5)
C3—C2—C1	121.5 (5)	C16'—C14—C17	75.4 (6)
C4—C3—C2	122.4 (4)	C15—C14—C17	104.6 (6)
C4—C3—H3	118.8	C14—C15—H15A	109.5
C2—C3—H3	118.8	C14—C15—H15B	109.5
C5—C4—C3	119.6 (4)	C14—C15—H15C	109.5
C5—C4—H4	120.2	C14—C16—H16A	109.5
C3—C4—H4	120.2	C14—C16—H16B	109.5
C4—C5—C6	119.0 (4)	C14—C16—H16C	109.5
C4—C5—N1	122.9 (4)	C14—C17—H17A	109.5
C6—C5—N1	118.1 (4)	C14—C17—H17B	109.5
C7—C6—C5	119.8 (4)	C14—C17—H17C	109.5
C7—C6—H6	120.1	C14—C15'—H15D	109.5
C5—C6—H6	120.1	C14—C15'—H15E	109.5
C6—C7—C2	122.3 (4)	H15D—C15'—H15E	109.5
C6—C7—H7	118.9	C14—C15'—H15F	109.5
C2—C7—H7	118.9	H15D—C15'—H15F	109.5
C9—C8—C13	122.3 (4)	H15E—C15'—H15F	109.5
C9—C8—O2	118.6 (4)	C14—C16'—H16D	109.5
C13—C8—O2	119.0 (4)	C14—C16'—H16E	109.5
C8—C9—C10	118.4 (4)	H16D—C16'—H16E	109.5
C8—C9—H9	120.8	C14—C16'—H16F	109.5
C10—C9—H9	120.8	H16D—C16'—H16F	109.5
C11—C10—C9	120.5 (4)	H16E—C16'—H16F	109.5
C11—C10—H10	119.8	C14—C17'—H17D	109.5
C9—C10—H10	119.8	C14—C17'—H17E	109.5
C12—C11—C10	119.7 (4)	H17D—C17'—H17E	109.5
C12—C11—H11	120.1	C14—C17'—H17F	109.5
C10—C11—H11	120.1	H17D—C17'—H17F	109.5
C11—C12—C13	121.5 (5)	H17E—C17'—H17F	109.5
C11—C12—H12	119.2	C5—N1—P1	130.2 (3)
C13—C12—H12	119.2	C5—N1—H1	114.9
C8—C13—C12	117.6 (4)	P1—N1—H1	114.9
C8—C13—H13	121.2	C14—N2—P1	126.0 (3)
C12—C13—H13	121.2	C14—N2—H2	117.0
C16—C14—C15'	71.0 (7)	P1—N2—H2	117.0
C16—C14—C17'	135.3 (6)	C8—O2—P1	118.8 (3)
C15'—C14—C17'	111.8 (6)	O1—P1—O2	115.41 (16)
C16—C14—N2	114.0 (5)	O1—P1—N2	117.03 (17)
C15'—C14—N2	111.4 (4)	O2—P1—N2	96.76 (17)
C17'—C14—N2	106.2 (5)	O1—P1—N1	107.61 (19)
C15'—C14—C16'	110.4 (7)	O2—P1—N1	107.01 (16)
C17'—C14—C16'	109.5 (6)	N2—P1—N1	112.47 (17)
			
C7—C2—C3—C4	0.3 (6)	C6—C5—N1—P1	159.1 (3)
C1—C2—C3—C4	179.6 (4)	C16—C14—N2—P1	32.5 (6)
C2—C3—C4—C5	0.7 (6)	C15'—C14—N2—P1	−45.5 (7)
C3—C4—C5—C6	−1.2 (5)	C17'—C14—N2—P1	−167.5 (5)
C3—C4—C5—N1	179.3 (3)	C16'—C14—N2—P1	75.5 (6)
C4—C5—C6—C7	0.7 (6)	C15—C14—N2—P1	−90.4 (6)
N1—C5—C6—C7	−179.8 (3)	C17—C14—N2—P1	155.9 (6)
C5—C6—C7—C2	0.3 (6)	C9—C8—O2—P1	−101.8 (4)
C3—C2—C7—C6	−0.8 (6)	C13—C8—O2—P1	80.5 (5)
C1—C2—C7—C6	179.9 (4)	C8—O2—P1—O1	−51.6 (3)
C13—C8—C9—C10	−1.0 (7)	C8—O2—P1—N2	−175.9 (3)
O2—C8—C9—C10	−178.6 (4)	C8—O2—P1—N1	68.1 (3)
C8—C9—C10—C11	0.0 (8)	C14—N2—P1—O1	54.8 (4)
C9—C10—C11—C12	1.2 (8)	C14—N2—P1—O2	177.8 (3)
C10—C11—C12—C13	−1.4 (8)	C14—N2—P1—N1	−70.6 (4)
C9—C8—C13—C12	0.7 (7)	C5—N1—P1—O1	−170.6 (3)
O2—C8—C13—C12	178.3 (4)	C5—N1—P1—O2	64.8 (3)
C11—C12—C13—C8	0.5 (8)	C5—N1—P1—N2	−40.3 (4)
C4—C5—N1—P1	−21.4 (5)		

### Hydrogen-bond geometry (Å, °)

**Table:**

*D*—H···*A*	*D*—H	H···*A*	*D*···*A*	*D*—H···*A*
N1—H1···N2^i^	0.88	2.32	3.175 (5)	163.
N2—H2···O1^ii^	0.88	2.40	3.275 (5)	170.

Symmetry codes: (i) −*x*+1/2, *y*+1/2, −*z*+1/2; (ii) −*x*+1/2, *y*−1/2, −*z*+1/2.
